# Modulation of EGR1 Expression by Hyperglycemia in Swine Rotator Cuff Tendons

**DOI:** 10.26502/josm.511500212

**Published:** 2025-07-17

**Authors:** Joey Day, Resmi Rajalekshmi, Devendra K. Agrawal

**Affiliations:** Department of Translational Research, College of Osteopathic Medicine of the Pacific, Western University of Health Sciences, Pomona, California, 91766, USA

**Keywords:** Diabetes, Early growth response-1, Hyperglycemia, Inflammation, Rotator cuff tendon, Stress granules, Toll-like receptor

## Abstract

Diabetes mellitus is known to impair tendon structure and function, yet the molecular mechanisms linking hyperglycemia to tendon degeneration remain poorly understood. This study investigated the expression of early growth response-1 (EGR1) and its association with toll-like receptor 4 (TLR4) and nuclear factor kappa B (NF-κB) signaling pathways in the rotator cuff tendons of hyperglycemic swine, a model chosen for its anatomical similarity to humans. Rotator cuff tendon tissues were collected from normal and hyperglycemic swine and analyzed using histology, qRT-PCR, Western blotting, and immunohistochemistry. Histological evaluation revealed altered tenocyte morphology and increased cellularity in hyperglycemic tendons. qRT-PCR results showed significant transcriptional upregulation of EGR1, TLR4, and NF-κB in hyperglycemic samples, suggesting activation of inflammatory and stress-response pathways. However, protein analysis revealed a non-significant decrease in EGR1 levels and modest increases in TLR4 and NF-κB, indicating possible post-transcriptional regulation. This discrepancy between mRNA and protein levels of EGR1 may be attributed to altered stress granule dynamics under hyperglycemic conditions. These findings elucidate a novel interplay among metabolic stress, innate immune signaling, and translational regulation in tendon tissue, proposing that targeting TLR4 signaling or stress granule formation may offer therapeutic potential for preserving tendon integrity in diabetic patients.

## 1. Introduction

The rotator cuff, composed of the supraspinatus, infraspinatus, teres minor, and subscapularis tendons, serves as the primary stabilizer of the glenohumeral joint—the most mobile joint in the human body [1–4]. This high degree of mobility makes the rotator cuff particularly susceptible to injuries such as tendinopathy, impingement, tears, and inflammation which can lead to significant pain, remodeling of extracellular matrix, fatty infiltration on tendons and muscles in rotator cuff lesions, and changes in mitochondrial activity [5–15]. All these cellular and molecular alterations may result in functional impairment due to decreased biomechanical properties of the rotator cuff, and increased morbidity. The prevalence of these injuries rises with age, ranging from 9.7% in individuals under 20 to 62% in those aged 80 and older [16]. Increased incidence of metabolic syndrome, hyperlipidemia, and other conditions are serious risk factors in the rotator cuff tears and re-injuries following the surgical repair of shoulder rotator cuff [17–21].

Hyperglycemia, a defining characteristic of diabetes mellitus (DM), results from insulin resistance and leads to chronically elevated blood glucose levels. DM is a global health concern, expected to affect nearly 300 million people by 2025 [22]. Sustained hyperglycemic conditions have been shown to alter RAGE-signaling pathways and other intracellular alterations in the tenocytes that disrupt extracellular matrix (ECM) remodeling, a key process in tendon healing, thereby potentially impairing recovery from rotator cuff injuries [23–25]. Given the high co-occurrence of diabetes and rotator cuff pathology, it is critical to explore the molecular mechanisms that link these conditions.

One molecule of particular interest is early growth response-1 (EGR1), a zinc finger transcription factor involved in regulating cell growth, differentiation, and survival across various tissues [26,27]. EGR1 plays a vital role in tendon biology by promoting tenogenic differentiation and enhancing collagen synthesis through transcriptional activation of Col1a1 and Col1a2 [28]. Inflammatory signaling pathways, particularly those involving tolllike receptor 4 (TLR4) and nuclear factor kappa B (NF-κB), may interact with EGR1 expression during tendon healing. Interleukin-1 (IL-1), released during the inflammatory phase, activates TLR4 and triggers NF-κB signaling, which promotes further IL-1 expression in a positive feedback loop [29]. Importantly, IL-1 has been reported to suppress EGR1 gene expression, suggesting that excessive or prolonged inflammation may hinder tendon repair by downregulating key regenerative factors [30].

Despite these insights, the impact of hyperglycemia on the expression of EGR1, TLR4, and NF-κB in rotator cuff tendons remains unclear. To address this gap, the present study utilizes a swine model—selected for its anatomical and physiological similarity to human tendon tissue [31] to assess the differential expression of these molecules in normal and hyperglycemic states. By examining these molecular changes, we aim to better understand how hyperglycemia affects tendon biology and to identify potential targets for improving rotator cuff healing in diabetic conditions.

## 2. Materials and Methods

### 2.1 Rotator cuff tendon tissue collection and preparation

The experimental research protocols (R21IACUC021 and R22IACUC034) were approved by the Institutional Animal Care and Use Committee (IACUC) of Western University of Health Sciences, Pomona, CA, USA. Yucatan miniswine (*Sus scrofa*) were obtained from Premier Bioresources (Ramona, CA, USA) and maintained under a 12hour light/dark cycle with twice-daily feeding. The animals were randomly assigned to two groups (n = 6 per group): normal and hyperglycemic. The normal group received a standard swine diet, while the hyperglycemic group was provided with a diet designed to induce hyperglycemia over a 6-month period. Blood glucose measurements confirmed group separation, with the normal swine exhibiting levels between 86–105 mg/dL and the hyperglycemic group ranging from 125–326 mg/dL. After 40 weeks, animals were euthanized, and infraspinatus tendon tissues were collected. Samples were preserved in 10% formalin for histological evaluation, stored in RNAlater for RNA extraction, or snap frozen at −80°C for subsequent protein analysis.

### 2.2 Histology Processing and Staining

Rotator cuff infraspinatus tendon (RCT) samples were processed using a Tissue-Tek VII system, involving sequential immersion in ethanol, xylene, and paraffin. Paraffin-embedded tissues were sectioned at 7 μm using a Leica RM2265 microtome and mounted on glass slides, followed by incubation at 60°C for one hour. Prior to staining, sections were deparaffinized and rehydrated. Hematoxylin and eosin (H&E) staining was performed using standard protocols [32]. Slides were mounted with Cytoseal 60 and imaged using a Leica DM6 light microscope at 100 μm scale. Three adjacent sections and 3–5 fields per section were analyzed.

### 2.3 Quantitative Real Time Polymerase Chain Reaction (qRT-PCR)

Approximately 50 mg of RCT tissue per sample was used for total RNA extraction using TRIZOL, following the manufacturer’s protocol. RNA concentration was measured with a Nanodrop 2000 spectrophotometer. Two micrograms of RNA were reversetranscribed into cDNA using the AzuraQuant^™^ cDNA Synthesis Kit and diluted 1:20 with nuclease-free water. RT-qPCR was performed in triplicate using AzuraView^™^ GreenFast qPCR Mix on a Bio-Rad thermal cycler under standard cycling conditions. Gene expression was normalized to 18S and analyzed using the ^ΔΔ^CT method (Table 1).

### 2.4 Western blot (WB)

RCT tissue (200 mg) was homogenized in RIPA buffer with protease inhibitors. After centrifugation, protein concentration in the supernatant was measured using the Bradford assay. Equal amounts of protein (25 μg) were separated by SDS-PAGE, transferred to PVDF membranes, and confirmed with Ponceau Red staining. Membranes were blocked, incubated with primary antibodies overnight at 4°C, followed by secondary antibody incubation. Protein detection was performed using ECL substrate and visualized with the ChemiDoc XRS+ System. Band intensities were quantified using Fiji (ImageJ), with 14–33ζ used as a loading control [33].

### 2.5 Immunohistochemistry (IHC)

Paraffin-embedded tissue sections were deparaffinized, rehydrated, and subjected to antigen retrieval using 1% citrate buffer heated for 15 minutes. After cooling and PBS washes, endogenous peroxidase activity was blocked with 3% hydrogen peroxide. Sections were then blocked with Normal Goat Serum for 1 hour and incubated overnight at 4°C with primary antibodies. The next day, slides were treated with biotinylated secondary antibodies, followed by VECTASTAIN^®^ ABC-HRP incubation and AEC substrate for color development. Hematoxylin (1:4) was used for counterstaining, and slides were mounted with Advantage mounting media. Semi-quantitative analysis of staining intensity and area was performed on three random images per section using Fiji (ImageJ) software [34] (Table 2).

### 2.6 Statistics

Statistical analysis was conducted using GraphPad Prism 10 for Windows (version 10.1.1). Data are expressed as mean ± standard deviation. Comparisons between the normal and hyperglycemic groups across all swine were evaluated using the nonparametric Mann-Whitney U test. A p-value less than 0.05 was considered statistically significant. For pairwise comparisons, significance levels were indicated as *p < 0.05, **p < 0.01.

## 3. Results

### 3.1 Histological analysis

**Figure 1:**
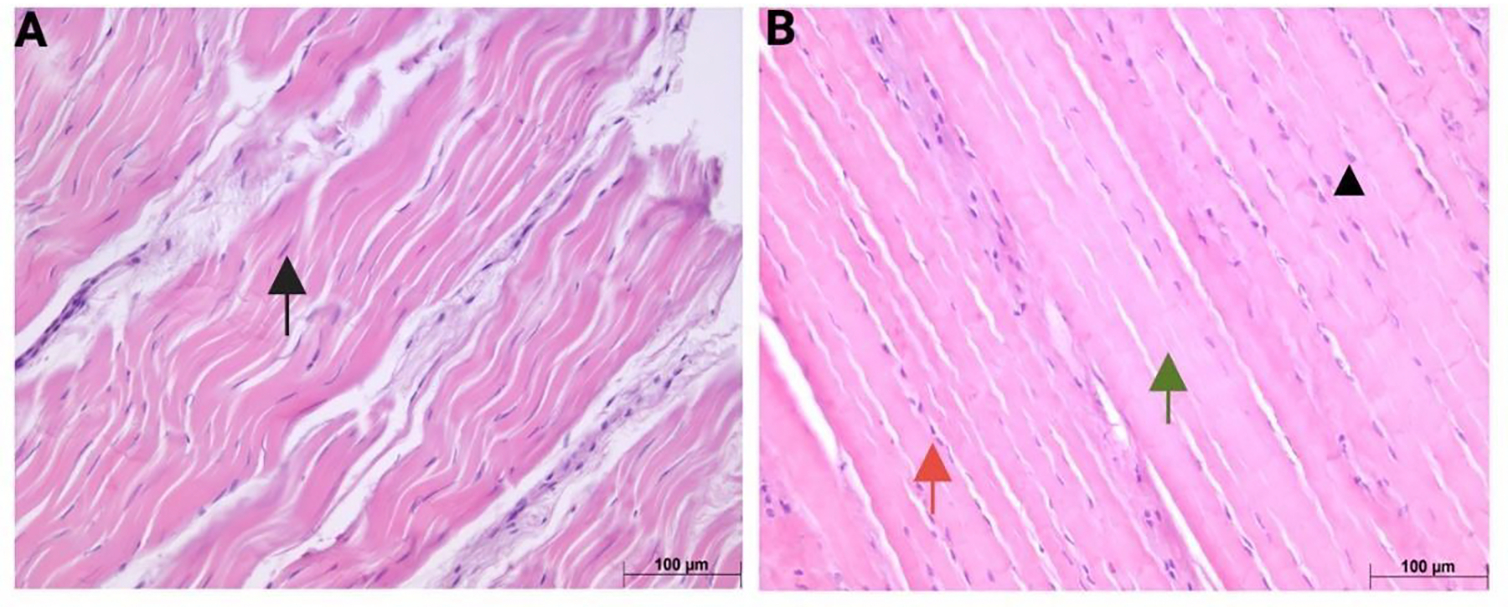
Representative hematoxylin and eosin (H&E) stained sections of rotator cuff tendons from normoglycemic (A) and hyperglycemic (B) swine. Black arrows indicate elongated tenocyte nuclei; arrowhead indicates rounded tenocyte nuclei; green arrows denote regions with absent or poorly visible nuclei; red arrows highlight areas of increased cellularity. Images are representative of all histology analyses for n = 6 (Normal swine) and n = 6 (Hyperglycemic swine).

Representative hematoxylin and eosin (H&E) stained sections of rotator cuff tendons from normal and hyperglycemic swine are presented in Figure 1A and 1B, respectively. In the normal group, tendon histoarchitecture was consistent with healthy tissue morphology, characterized by densely packed, well-organized collagen fibers aligned in a parallel orientation. Tenocytes exhibited elongated, spindle-shaped nuclei (denoted by black arrows), which were uniformly aligned along the axis of the collagen fibers and embedded within a homogenous and structurally intact ECM.

In contrast, tendons from the hyperglycemic group demonstrated distinct morphological alterations. Although the general alignment of collagen fibers appeared preserved, several degenerative features were observed. Tenocyte nuclei exhibited a loss of their characteristic elongated morphology, appearing more rounded or oval (arrowhead), and in certain regions, nuclei were absent or poorly visible. (green arrow). Additionally, there was a prominent increase in cellularity (red arrow).

### 3.2 Hyperglycemia-Induced Upregulation of EGR1, TLR-4, and NF-κB in Rotator Cuff Tendons

To evaluate the impact of hyperglycemia on the transcriptional regulation of EGR1, TLR-4, and NF-κB in rotator cuff tendons, qRT-PCR was performed. Relative gene expression levels were calculated using the ^ΔΔ^Ct method, normalizing to 18S rRNA as the endogenous control and comparing against normal controls.

**Figure 2:**
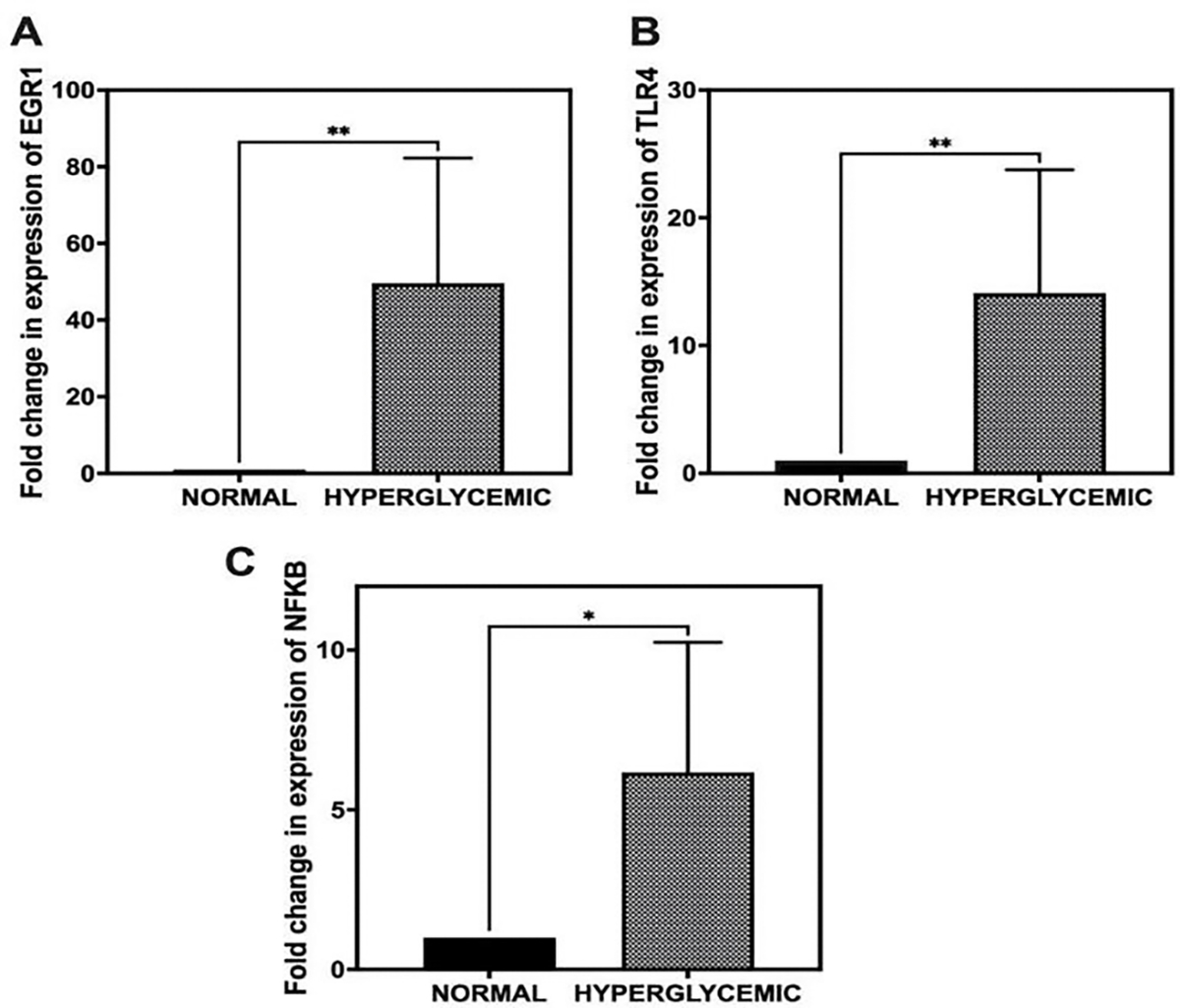
Quantitative real-time PCR (qRT-PCR) analysis of gene expression levels in rotator cuff tendons under hyperglycemic and normoglycemic conditions. (A) Expression of EGR1. (B) Expression of TLR-4. (C) Expression of NF-κB. Gene expression was normalized to 18S rRNA and calculated using the ^ΔΔ^Ct method. Data are presented as mean ± SD. n = 6 for normal swine and n = 6 for hyperglycemic swine. *p < 0.05, **p < 0.01.

The results demonstrated a significant upregulation of all three target genes in the hyperglycemic group. EGR1 expression exhibited a substantial increase, with a fold change of 49.64 ± 32.67 relative to the normal group (Figure 2A). Similarly, TLR-4 expression was elevated by 14.11 ± 9.67-fold, while NF-κB showed a 6.17 ± 4.07-fold increase in expression (Figure 2B, 2C). These findings indicate a strong transcriptional response of these genes under hyperglycemic conditions in tendon tissue.

### 3.3. Western Blot Analysis Reveals Trends in EGR1 Expression in RCT Under Hyperglycemia

**Figure 3:**
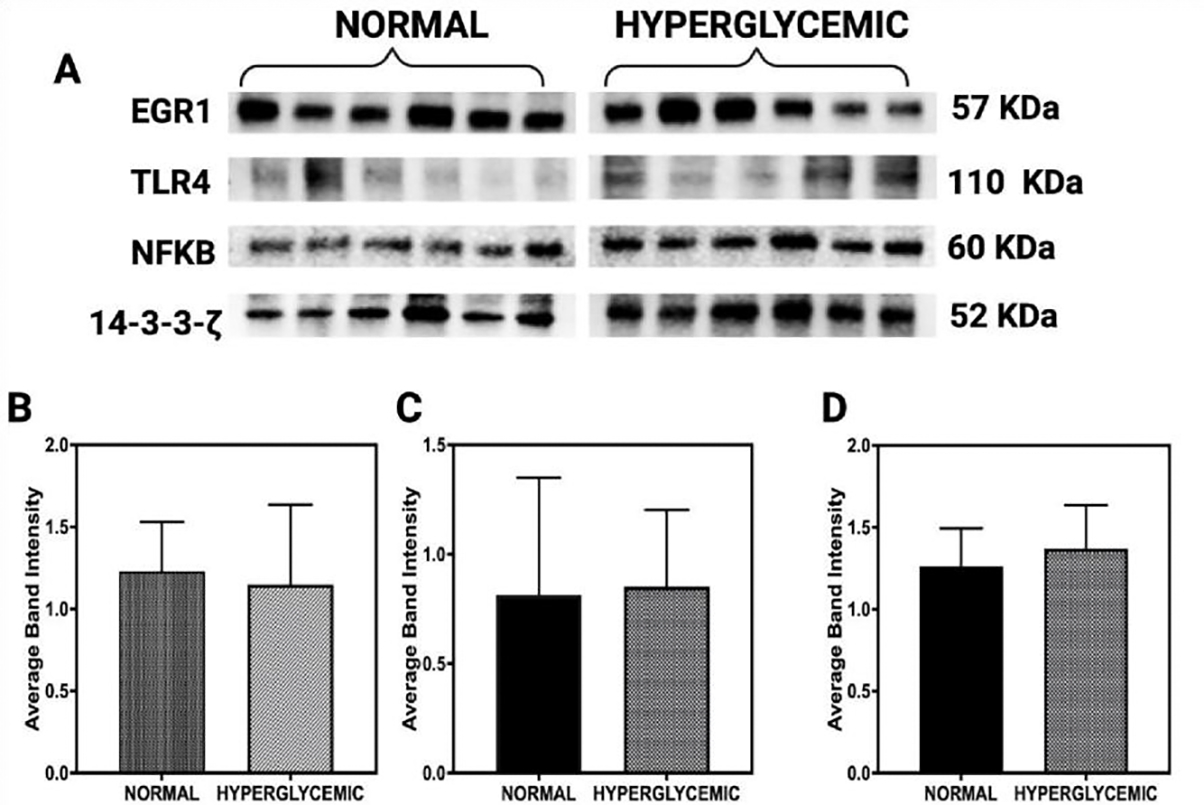
Western blot analysis of protein expression in rotator cuff tendon lysates from normoglycemic and hyperglycemic swine. (A) Representative immunoblots for early growth response protein 1 (EGR1; 57 kDa), Toll-like receptor 4 (TLR4; 110 kDa), nuclear factor kappa B (NF-κB; 60 kDa), and 14–3-3-ζ (52 kDa) as a loading control. (B–D) Densitometric quantification of EGR1, TLR4, and NF-κB normalized to 14–3-3-ζ. Data are presented as mean ± SD. Sample sizes: n = 6 for normal swine and n = 6 for hyperglycemic swine.

Western blot analysis was performed to evaluate the expression levels of early growth response protein 1 (EGR1; 57 kDa), Toll-like receptor 4 (TLR4; 110 kDa), and nuclear factor kappa B (NF-κB; 60 kDa) in rotator cuff tendon lysates obtained from normoglycemic and hyperglycemic swine (Figure 3A). The protein 14–3-3-ζ (52 kDa) served as an internal loading control for normalization [31].

Densitometric quantification demonstrated a reduction in EGR1 expression in tendons from hyperglycemic swine compared to normoglycemic controls (Figure 3B). However, this decrease did not reach statistical significance (p>0.05), indicating a potential trend rather than a conclusive downregulation. In contrast, TLR4 expression showed a mild increase in the hyperglycemic group (Figure 3C), aligning with its established role in initiating innate immune responses during metabolic stress. Similarly, NF-κB levels, a downstream target of TLR4 signaling and a central mediator of inflammatory pathways, exhibited a slight upregulation under hyperglycemic conditions (Figure 3D).

### 3.4 Immunohistochemistry shows stable EGR1, TLR4, and NF-κB levels despite hyperglycemia

**Figure 4:**
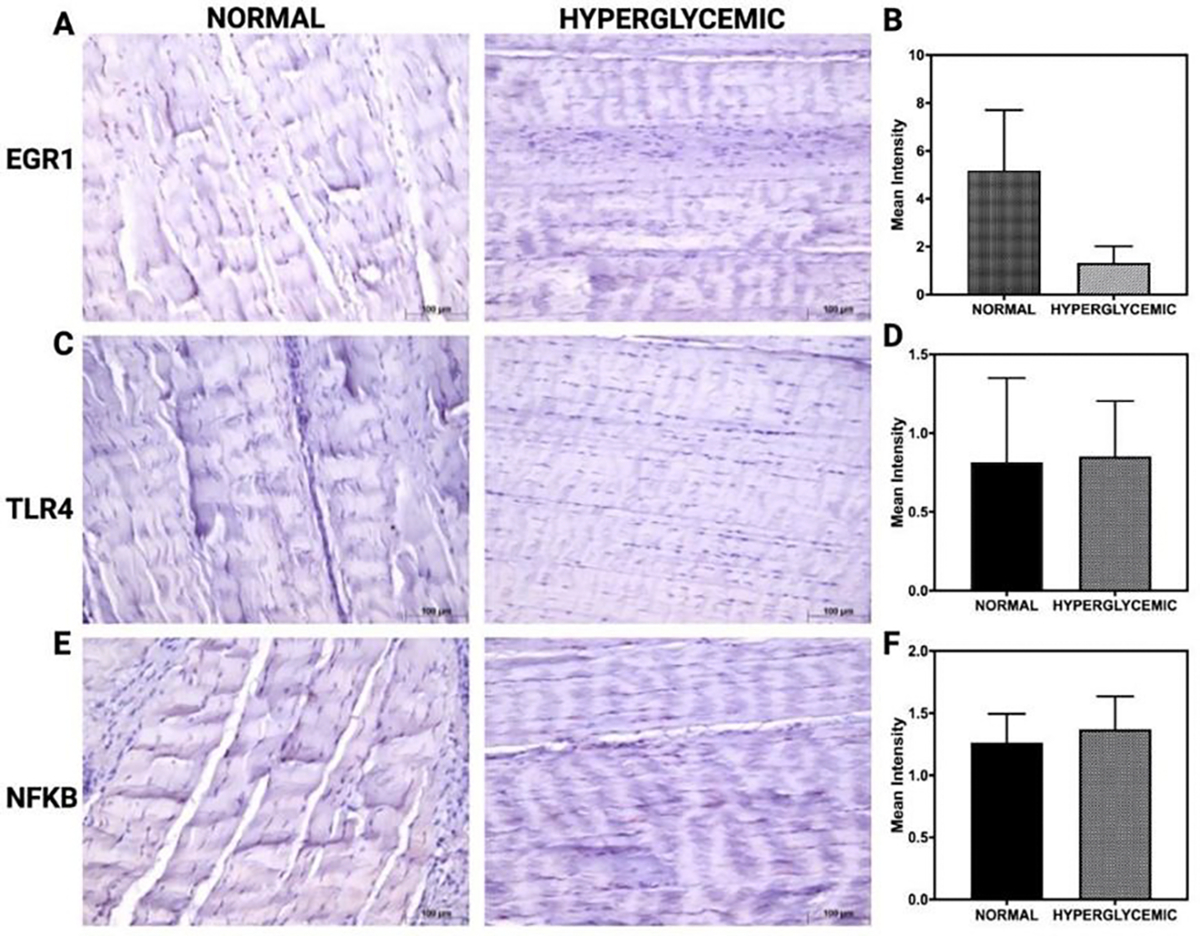
Immunohistochemical analysis of EGR1, TLR4, and NF-κB expression in RCT under normoglycemic and hyperglycemic conditions. (A, C, E) Representative IHC images of tendon tissue sections stained for EGR1 (A), TLR4 (C), and NF-κB (E) in normoglycemic (left) and hyperglycemic (right) groups. (B, D, F) Quantification of mean staining intensity for EGR1 (B), TLR4 (D), and NF-κB (F). Data are presented as mean ± SD. Images are representative of all IHC analyses for n = 6 for normal swine and n = 6 for hyperglycemic swine.

Figure 4 represents immunohistochemical (IHC) staining of rotator cuff tendon tissues evaluating the expression of EGR1, TLR4, and NF-κB under normal and hyperglycemic conditions. Images comparing EGR1 staining between normal and hyperglycemic groups reveal a qualitative reduction in signal intensity under hyperglycemia (Figure 4A). However, the corresponding quantitative analysis shows no statistically significant difference in mean intensity, as reflected by overlapping error bars and substantial inter-sample variability (Figure 4B). For TLR4, immunostaining results show no visible alteration in expression between groups (Figure 4C). Quantification confirms that hyperglycemia does not induce a significant change in TLR4 protein levels (Figure 4D). Similarly, the expression of NF-κB remains consistent across both conditions, with no significant differences observed in staining intensity measurements (Figure 4E and 4F).

## 4. Discussion

The present study provides compelling evidence that hyperglycemia induces significant morphological and molecular alterations in rotator cuff tendon tissues, implicating key stress and immune signaling pathways. Histological analyses revealed degenerative changes in hyperglycemic tendons, including altered tenocyte morphology and increased cellularity, which are hallmarks of tendon pathology. At the molecular level, qRT-PCR analysis showed a robust upregulation of *EGR1*, *TLR4*, and *NF-κB* transcripts under hyperglycemic conditions, highlighting a transcriptional activation of inflammatory and stress-responsive pathways. However, protein-level data from Western blot and IHC revealed more subtle alterations, including a non-significant decrease in EGR1 protein and modest changes in TLR4 and NF-κB. These findings suggest the involvement of complex post-transcriptional or translational regulatory mechanisms.

TLR4 signaling emerges as a central mediator in the hyperglycemia-induced tendon pathology observed in this study. TLR4, a pattern recognition receptor known for its role in innate immunity, is activated under metabolic stress conditions such as diabetes. Once activated, TLR4 initiates downstream signaling cascades involving MyD88/TRIF ultimately leading to the activation of NF-κB, a transcription factor pivotal for the expression of pro-inflammatory cytokines and stress response genes [35,36]. In our findings, the mild upregulation of TLR4 and NF-κB proteins in hyperglycemic tendons aligns with this known function, suggesting that chronic metabolic stress primes tendon cells toward an inflammatory phenotype.

Importantly, EGR1, a zinc-finger transcription factor, plays a crucial role in tendon development and repair by regulating genes involved in ECM synthesis and cell survival [27]. High transcriptional levels of EGR1 observed via qRT-PCR contrast with its non-significantly reduced protein expression, pointing toward possible post-transcriptional regulation. This discrepancy gains further importance in the context of cellular stress responses, specifically stress granule (SG) dynamics.

Stress granules are cytoplasmic aggregates of stalled translation pre-initiation complexes that form in response to cellular stress and help maintain homeostasis by selectively sequestering mRNAs, thereby modulating their translation [37]. Under hyperglycemic conditions, oxidative stress can trigger SG formation, influencing the fate of specific transcripts, including *EGR1*. A recent study has shown that stress granules can sequester *EGR1* mRNA in response to chemotherapy-induced stress, leading to reduced EGR1 translation and attenuated apoptosis [38]. Applying this concept to tendon biology, under hyperglycemic oxidative stress, EGR1 mRNA is likely sequestered in stress granules, leading to reduced protein levels despite high mRNA expression. This negatively impacts tendon healing by lowering EGR1 availability, which is crucial for promoting ECM production and supporting cellular repair. In line with this, our study observed increased oxidative stress in hyperglycemic tendon tissue, as evidenced by DHE staining, and a concurrent upregulation of the stress granule marker G3BP1 protein, although these findings were not shown in the current manuscript.

Moreover, the role of TLR4 in regulating SG dynamics introduces another layer of complexity. It has been demonstrated that activation of TLRs, including TLR4, can inhibit SG assembly via the IKK complex pathway in a ligand-dose dependent manner [39]. This inhibition of SG formation could initially prevent the sequestration of critical mRNAs like *EGR1*, allowing transient translation. However, persistent activation of TLR4 signaling under chronic hyperglycemia might lead to dysregulated SG dynamics, either through premature disassembly or excessive inhibition, disturbing the delicate balance of translation control necessary for tendon homeostasis.

The integration of these findings suggests a complex regulatory network in hyperglycemic tendons, where the activation of TLR4 and the dynamics of stress granules converge to influence the availability of EGR1. Since EGR1 is typically beneficial for tendon health by promoting anabolic repair processes, its reduced presence due to altered stress granule behavior or translational inhibition could contribute to the tendon degeneration observed in diabetic tendinopathy. Therefore, therapeutic strategies that target TLR4 signaling or stress granule formation may provide new opportunities for improving tendon health in diabetic patients.

Inhibiting the TLR4–IKK–NF-κB pathway may help restore normal stress granule (SG) dynamics, which in turn would preserve the translation of EGR1. Additionally, exploring targeted modulation of SG components could prevent the harmful sequestration of EGR1 mRNA during oxidative stress. Gene therapy aimed at increasing EGR1 protein levels, as well as small molecules that enhance EGR1 translation, could also be promising strategies for mitigating diabetic tendon degeneration.

## 5. Conclusion

In conclusion, this study demonstrates that hyperglycemia induces significant histological and molecular alterations in rotator cuff tendons, characterized by degenerative cellular changes and a pronounced upregulation of EGR1, TLR4, and NFκB transcripts. The discrepancy between the increased mRNA levels and the stable or decreased protein expression—especially for EGR1—indicates the involvement of intricate post-transcriptional regulatory mechanisms, likely driven by stress granule dynamics affected by oxidative stress and TLR4 signaling. These findings reveal a complex regulatory network in diabetic tendon pathology, in which chronic metabolic stress interferes with normal tendon repair by altering critical inflammatory and regenerative pathways. Targeting TLR4 signaling and stress granule pathways may thus represent promising therapeutic strategies to restore tendon homeostasis and improve healing outcomes in diabetic patients.

## 6. Limitations and Future Prospects

A limitation of this study is that swine samples were collected at a single time point, and the animals were subjected to a hyperglycemic diet for only six months. To gain a more comprehensive understanding of EGR1 expression in response to hyperglycemia, future studies should include multiple time points. A longitudinal approach would allow for the evaluation of EGR1 regulation across various stages of disease progression, the identification of critical phases in the inflammatory response, and the characterization of temporal dynamics in the expression of upstream regulatory molecules. This would offer deeper insight into the progressive modulation of EGR1, its role in extracellular matrix remodeling, collagen deposition, and the overall impact on tendon integrity and inflammation under chronic hyperglycemic conditions.

## Figures and Tables

**Table 1:** Forward and reverse nucleotide sequence of primers used for qRT-PCR in this study.

Gene Name	Forward Nucleotide Sequence	Reverse Nucleotide Sequence
TLR4	5’-GGGTCATGCTTTCTCCGGGT-3’	5’-TTTCACATCTGCACGCAAGGG-3’
EGR1	5’-ACAGTGGTTTCCCATCACCCTC-3’	5’-GTTGGTGACAGCTGAGGAAGGA-3’
NF-κB	5′-GACTACGACCTGAATGCTGTG-3′	5’-GTC AAA GATGGG ATG AGG AAGG-3′
18S	5’-CCCACGGAATCGAGAAAGAG-3’	5’-TTGACGGAAGGGCACCA-3’

**Table 2:** Primary and secondary antibodies used for WB and IHC.

Antibody	Supplier	Catalog #	Dilution in WB	Dilution in IHC
**Primary Antibodies**				
TLR4	Proteintech	66350-1-Ig	1:2000	1:50
EGR1	Santa Cruz	sc-515830	1:100	1:50
NF-κB	Proteintech	10745-1-AP	1:3000	1:100
14-3-3ζ	Mybiosource.com	MBS9601925	1:1000	-
**Secondary Antibodies**				
Anti-mouse	Novus biologicals	NB7544	1:3000	-
Anti-rabbit	Thermofisher scientific	A16023	1:2000	-
Anti-mouse	Vector Laboratories	BP-2000-50	-	Ready-to-use
Anti-rabbit	Vector Laboratories	BP-9100-50	-	

## Data Availability

Data will be made available on request.
